# Prognostic Significance of Immunohistochemical Phenotypes in Patients Treated for High-Grade Cervical Intraepithelial Neoplasia

**DOI:** 10.1155/2013/831907

**Published:** 2013-12-18

**Authors:** Massimo Origoni, Marta Parma, Giacomo Dell'Antonio, Chiara Gelardi, Chiara Stefani, Stefano Salvatore, Massimo Candiani

**Affiliations:** ^1^Department of Gynecology & Obstetrics, Vita-Salute San Raffaele University, School of Medicine at San Raffaele Scientific Institute, Via Olgettina 58, 20132 Milan, Italy; ^2^Department of Pathology, Vita-Salute San Raffaele University, School of Medicine at San Raffaele Scientific Institute, Via Olgettina 58, 20132 Milan, Italy

## Abstract

Strong evidence exists that the host's immune system plays a crucial role for the development of human papillomavirus-related cervical premalignant and malignant lesions. In particular, effective cell-mediated immunity (CMI) promotes spontaneous infection clearance and cancer precursors regression in healthy subjects, while immunosuppressed individuals are more likely to experience infection persistence, cervical intraepithelial neoplasia (CIN) lesions, and cervical cancer. In this study, the prognostic significance of immunohistochemical profiling of CD4+ T-cells, CD8+ T-cells, dendritic cells (CD11c+), T-bet+, and GATA-3+ transcription factors has been studied in surgical specimens of 34 consecutive women affected by high-grade cervical intraepithelial neoplasia (CIN2-3) submitted to cervical conization. Results have been correlated with the clinical outcomes at 24 months after treatment and statistically analyzed. Higher rates of CD4+ T-cells, CD11c+ dendritic cells, and T-bet+ transcription factor positivity showed a strong statistically significative correlation with favourable clinical outcomes (*P* ≤ 0.0001). These data reinforce the evidence of the relevance of the host's immune status in the natural history of HPV-related cervical disease and add a prognostic significance of the cervical immunological profile in terms of predicting significant lower recurrence rates.

## 1. Introduction 

Uterine cervical cancer is the second most frequent gynecological malignancy worldwide [1] and high-risk human papillomavirus (hr-HPV) infection has been recognized as the major risk factor for the development of high-grade cervical intraepithelial neoplasia (CIN2-3) and invasive cancer [2–4]. It is well documented that most HPV genital infections and low-grade cervical intraepithelial neoplasia (HPV-CIN1) are transient and regress spontaneously over a period of several months, predominantly in younger patients [5, 6]; nonetheless, several longitudinal studies have indeed consistently reported that the persistence of HR-HPVs, together with other risk cofactors, have been implicated in the infection persistence and/or progression to more severe precancerous lesions. Sexual habits, cigarette smoking, and lack of screening tests are consistently identified as crucial risk factors [7]. Several studies also signify the prevalence of HPV infections and the importance of host immune response in the clearance of HPV infections [8–10]. Immunosuppressed women show an increased incidence of HPV infections, genital warts, and persisting CIN lesion [11]. In such individuals, like human immunodeficiency virus- (HIV-) infected women and organ transplant recipients, the prevalence of HPV infections and anogenital dysplasia/neoplasia is significantly higher than in the general population [12–16], immunosuppression with being strongly associated with persistence of HPV infections and subsequent progression to neoplastic transformation. Also, a considerable amount of work has been done with regard to immunoprofiling in invasive cervical cancer; consistently, different independent studies have demonstrated a positive prognostic effect of high counts of neutrophils and myeloid cells towards survival, lymph node metastases, and recurrences in cervical cancer patients [17–19]. The immune system is known to play a key role: both cell-mediated immunity (CMI) and antibody production have been implicated in influencing the persistence or clearance of genital HPV infection [9]. The first defense against HPV is represented by the innate immune system, which provides nonspecific protection against a variety of pathogens and enhances the adaptive immune response. However, HPV infections often evade innate immune recognition and elimination. HPV early gene expression and shedding of viral particles occur in the superficial layers of the squamous genital epithelium, where virus antigens are not readily detected and keratinocytes do not promote cellular lysis, so that no or very poor proinflammatory response is elicited [10]. For this, HPVs promote cell proliferation instead of lysis and do not determine a systemic infection; as a result, both HPV-specific CMI responses and antibody responses are very less effective than those elicited by many other viral pathogens [20, 21]; thus, the lower female genital tract epithelia behave as a protective microambient for the virus replication. Moreover, HPV infection can interfere with the local immune vigilance mechanism both in the induction phase (antigen presentation) and in the effector phase (generation of antibody-producing B cell and cytotoxic T cell) [10]. A polarization of the immune response for T-helper (Th) Th2 has been reported in women with HPV infections that progress to high-tgrade lesions [22, 23]. The immune evasion that characterizes HPV infection may eventually support the establishment of HPV persistent infection, leading to the development of cervical cancer [9, 10, 24]. Immunohistochemical studies of the expression of CD4+ T-cells (Th1) and of Th2-type cytokines show that cytokine-secreting cells are present in the subepithelial cervical tissue. Significantly lower percentages of Th1 cells with higher proportions of IL4+ and/or IL6+ secreting cells were observed in high-grade CINs compared to normal tissues. Predominant Th2-type cytokines and CD4+ T-cells were also found in high-grade cervical lesion [25]. Accordingly, several studies have shown that high CD4+ infiltrates are associated with the regression of genital warts [26–29]. With regard to these observations, in the present study, we focused our attention on the cervical immunity, investigating if an association between tissue immunological patterns and the outcome of patients undergoing cervical conization for a high-grade CIN existed. In our study, we looked at different aspects of the cervical immunoprofile: CD11c+ dendritic cells, CD4+ and CD8+ T-lymphocytes, T-bet+, and GATA-3+ transcription factors. Thus, the aim of the present study was to primarily investigate if a prognostic correlation between the tissue cell-mediated immune responses of the uterine cervix and the follow-up clinical outcome existed in patients conservatively treated with a surgical excisional procedure for a high grade (CIN2-3) cervical preneoplastic lesion.

## 2. Materials and Methods

### 2.1. Patients and Samples

Thirty-four consecutive patients, known for a previous conization with loop electrosurgical excision procedure (LEEP) or cold knife technique for a high-grade cervical preneoplastic lesion (15 cases of CIN2 and 19 cases of CIN3) clinically followed up at our institution, were selected and resulted eligible for this retrospective study. Exclusion criteria were HIV-positive patients, congenital and acquired immunodeficiency, pharmacological immunosuppression, pregnancy, and lower genital tract infections at the moment of surgical procedure (negative microbiological swab 7 days prior to procedure). Histological specimens were all reviewed by one pathologist. Postsurgical follow-up was scheduled according to accepted guidelines: cervical cytology (Pap test), high-risk HPV-DNA testing (Hybrid Capture 2 (HC2), Qiagen, USA), and colposcopy with cervical biopsy if indicated were performed at 6, 12, 18, and 24 months after the surgical procedures. According to the results of the performed tests at the end of follow-up, cases were identified as cured (negative follow-up) or relapsed (positive histology). Cases with persistence of cervical lesions (CIN) detected up to the second follow-up visit (12 months) were not included, in order to analyze the real recurrence rates.

### 2.2. Histology and Immunohistochemical Evaluation

**Figure 1:**
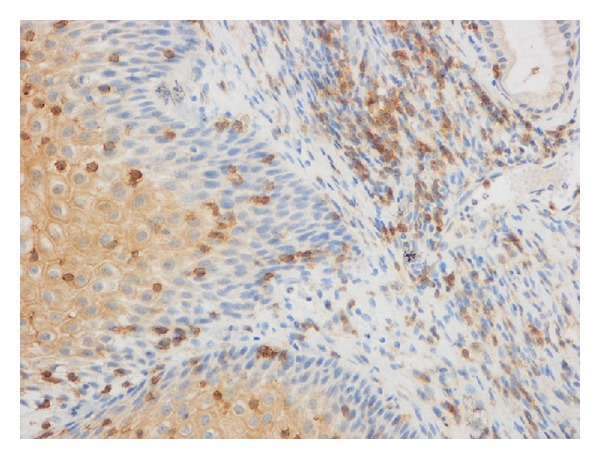
CD4+ T-cells immunohistochemical staining.

**Figure 2:**
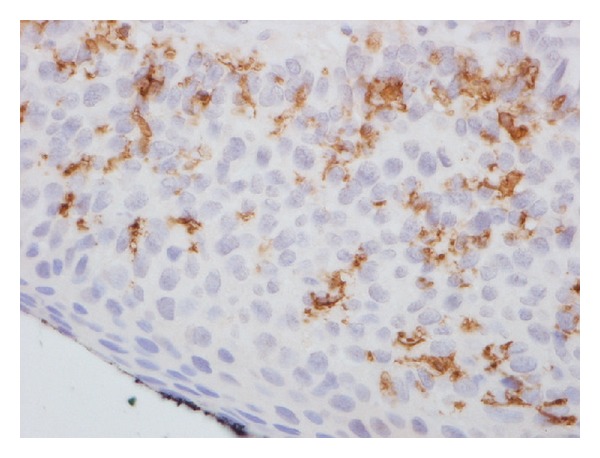
CD11c+ (dendritic cells) immunohistochemical staining.

**Figure 3:**
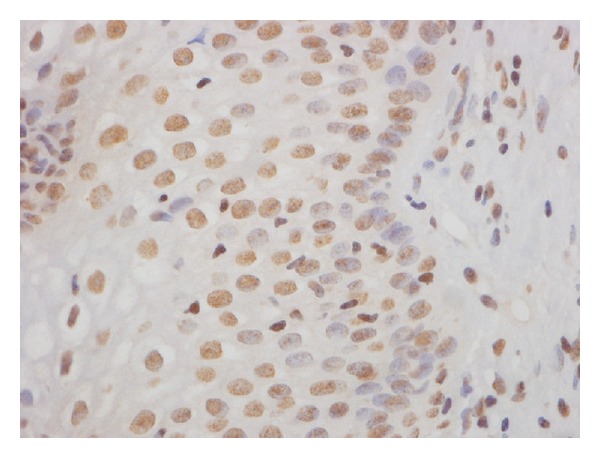
T-bet+ transcription factor immunohistochemical staining.

Paraffin-embedded histology blocks of the 34 studied cases were retrieved and, for each case, ten 4-micron sections were selected according to the presence of very significative areas of high-grade dysplastic lesions and inflammatory epithelial and stromal infiltrate. All slides were reviewed by the same pathologist. Immunohistochemistry was carried out on tissue sections with the following antibodies: (1) anti-CD4 (Novocastra Laboratories, UK) for T lymphocytes helper inducer, (2) anti-CD8 (Neo Markers, USA) for cytotoxic T cells, (3) anti-CD11c (Novocastra Laboratories, UK) for dendritic cells, (4) anti-T bet (Santa Cruz, USA) for the shift of Th1 immune response, and (5) anti-GATA3 (R&D Systems Inc., USA) for detecting the shift of an antibody immune responses. All immunostainings were performed on automated immunostainers using avidin-biotin peroxidase standard technique after antigene retrieval methods in a microwave oven. Final immunohistochemical evaluation was performed in a blind fashion and scored by the same experienced pathologist. Data were categorized into 4 classes, according to the percentage of inflammatory cells' infiltrates detected: 0. <2% of immunohistochemistry-positive inflammatory cells; 1. >2<5% of immunohistochemistry-positive inflammatory cells; 2. >5<10% of immunohistochemistry-positive inflammatory cells; 3. >10% of immunohistochemistry-positive inflammatory cells (Figures 1, 2, and 3). Scores of positivity have been correlated with clinical outcomes after follow-up completion (cured versus recurred) and statistically analyzed.

### 2.3. Statistical Analysis

For statistical purposes, scores of positivity have been aggregated into two variables: score 0: <5% of positivity and 1: >5% of positivity, respectively. Statistical analysis was performed with chi-squared, Fisher's exact, and Phi tests, assuming an alpha value <0.05 as significant. All statistical analyses were performed with SPSS 15.0 software for Windows.

### 2.4. Ethical Issues

Informed consent for scientific use of biological specimens was obtained for each included case as routine procedure at our institution. Due to study design and data collection, Institutional Review Board (IRB) gave ethical approval.

## 3. Results

The study group is represented by a total of 34 selected cases conservatively treated with cervical conization (LEEP or surgical) for CIN lesions (15 CIN2 and 19 CIN3); patients' mean age was 37 yrs (min. 20 max. 54 yrs). All cases had a histological diagnosis on the surgical specimen of squamous CIN2-3 with free surgical margins, confirmed after slides revision by the same pathologist. All cases preoperatively tested positive for HPV 16 and/or 18 with HC2 assay. At the end of the 24-month follow-up, 22 (64.2%) cases were identified as cured, while, in 12 (35.3%), a disease recurrence was detected; out of these, 8 (66.6%) cases showed a histologically proven CIN2 lesion and 4 (33.3%) a CIN3.

### 3.1. Correlations among Immunological Targets and Outcome

Scores of immunohistochemical positivity in the studied tissue sections have been correlated with the clinical outcomes of patients at the end of the follow-up period of 24 months and statistically analyzed. Results obtained from the epithelial and stromal evaluation are summarized in Tables 1 and 2, respectively. We demonstrated that the percentage of CD4+ T-lymphocytes detected either within the epithelium or the stroma significantly correlates with patients' outcomes (*P* < 0.0001); in particular, an inverse correlation between CD4+ T-lymphocytes infiltration and disease outcome (epithelium: Phi −0,70; stroma: Phi −0,83) has been observed. This means that for elevated positivity for CD4+ T-lymphocytes infiltration, a correlation with a statistically significative reduced prevalence of recurrences of the disease was demonstrated. The same association pattern, both for cervical epithelium and stroma, was observed for T-bet+ immunohistochemical staining (*P* < 0.0001); values obtained from the Phi test showed a significant inverse relationship between the variables (epithelium: Phi −0.78; stroma Phi −0.83). Again, higher values of positivity correlate with a significative lower incidence of disease recurrence. It is noteworthy to underline that, in cases where a >5% immunohistochemical positivity for both CD4+ T-cells and T-bet+ transcription factor was detected, either within the epithelial or the stromal cervical tissue, no cases of recurrent CIN were reported (*P* < 0.0001). With regard to the analysis of the positivity for CD8+ T-cells and GATA3+ transcription factor, both for the epithelium and for the stroma, no significative correlations with patients' outcome were obtained. A different behaviour has been observed for CD11c+ dendritic cells' immunohistochemical positivity: no significative correlation with patients' outcome was observed according to CD11c+ amounts of positivity within the epithelium, while a significative correlation existed for the stroma (*P* = 0.0001).

## 4. Discussion

Testing patients for human papillomavirus has recently emerged as the crucial step for an effective cervical cancer prevention strategy [30–34], but the evidence that HPV represents the necessary but not unique cause for the development of cervical cancer [35] has moved the attention to the potential cofactors responsible for the development of the disease. Among those that have been investigated, a significant role has been demonstrated for the host immune system [36]; in this view, the immune system has emerged as a double-sided effector of the host's response to HPV infection: from one side, the effectiveness of the immune response promotes the spontaneous clearance of the virus, while reduced immunity is correlated with the persistence of the virus and the onset/progression of preneoplastic lesions [37, 38]. Several lines of evidence suggest that both humoral and cell-mediated response have a key role in the HPV infection natural course. This is confirmed by the generally accepted correlation between high prevalence of viral infection and cervical cancer in immunocompromised women [39]. In this field, a higher prevalence of hr-HPV strains (especially 16 and 18) has been demonstrated in transplanted and HIV-positive women [11–16]. Despite these observations, the issue of considering immunosuppressed women as a homogeneous group of at-risk patients has recently been argued; we recently demonstrated that female transplant recipients, submitted to different regimens of drug immunosuppression, did not show an increased incidence/prevalence of hr-HPV infection or intraepithelial preneoplastic cervical lesions (CIN) compared to healthy controls during a 10-year gynecological follow-up [40]. In terms of mucosal innate immunity, it is known that cervical tissues present lots of dendritic and Langerhans cells. Dendritic cells are crucial, being involved in antigens processing; in fact, they migrate to regional lymphnodes for antigens presentation to B and T cells, determining the development of the acquired response [41]. Dendritic cells also have a primary role in creating a local proinflammatory environment: they upregulate class I and II major HLA complex and costimulators molecules as CD80 and CD86, promoting cytokines release; all these steps are at the basis of the activation of naïve CD8 and CD4 T cells in lymph nodes [42]. In patients affected by HPV-related cervical cancer, apoptotic cells originated by the epithelium turnover are phagocytosed by dendritic cells that release immunosuppressive cytokines as TGFb, IL10, and IL13 [22, 23]. In addition, the E7 viral oncoprotein inhibits INF-*α* release, reducing the inflammatory process in the infection site [24]. Through these processes, HPV infection prevents dendritic cells maturation and activation, reducing the effective T-cells response; moreover, a reduction of dendritic cells in the site of cervical cancer has been demonstrated, in contrast to what is observed in normal cervical tissue [43]. A recent study demonstrated that dendritic cells are cytotoxic towards HPV-related cancer cells; to evaluate dendritic cells and Langerhans cells cytotoxicity, normal cervical cells were sensitized towards HPV16 E6 and E7 proteins and cytotoxicity was measured in terms of production of proinflammatory cytokines; dendritic cells and Langerhans cells were demonstrated as equally cytotoxic towards HPV16 E6 and E7 proteins expressing cells [44]. With regard to the role of the systemic immunity in response to HPV infection, some discordant results can be underlined: in one study [45], CD4+ T-lymphocytes of patients with intraepithelial lesions did not react towards HPV16 peptides, while CD4+ T cells of patients with cervical cancer proliferated without cytokines production. In another study [46], CD4+ T cells of patients with low-grade lesions developed a Th1 response, while patients with cervical tumor had a Th2 response elicited. In our experience [47], specific CD4+ T cells towards the HPV18 E6 oncoprotein were detected in 80–90% of HPV18-positive patients affected by a high-grade CIN and a Th1 versus Th2 response was demonstrated; in addition, we showed that INF-*γ* production influenced the disease course. In fact, we observed that high levels of INF-*γ* correlated with lesions regression, while low cytokines levels were observed in patients with disease persistence. As far as it concerns the density and distribution of lymphocytes subpopulations in dysplastic HPV-related cervical tissues, the correlation with disease regression versus progression has already been underlined [25]; the authors immunohistochemically characterized T cells' phenotype in cervical biopsies of regressive and progressive intraepithelial neoplasia (CIN), demonstrating that regressive lesions showed a greater distribution of CD4+ T cells both in the stroma and in the epithelium, while progressive and/or high-grade lesions (CIN3) showed a higher CD4/CD8 ratio. As for dendritic cells and T-bet+ and GATA-3+ transcription factors, to our knowledge, no previous studies investigated their relevance in cervical dysplastic tissues (CIN) in terms of prognostic relevance after standard treatment, while GATA-3+ was already studied in cervical carcinogenesis [48]. Thus, our study design was aimed at the identification of possible correlations among innate/acquired immunity of the uterine cervix affected by high-grade intraepithelial neoplasia (CIN2-CIN3) and the rate of disease recurrences after surgical conservative treatment. In our series, CD4+ T cells distribution resulted closely associated with favourable patients' postsurgical outcomes (*P* < 0.0001) and the results of the tests of association show a strong inverse association between the variables; in fact, we obtained a Phi value of −0.70 (*P* < 0.01) and −0.83 (*P* < 0.01) for the epithelium and the stroma, respectively. Thus, higher values of CD4+ local immunity correlated with a significantly higher prevalence of regression of the disease after treatment. On the other end, CD8+ cytotoxic T-lymphocytes did not show a significant association with outcomes, therefore not emerging as relevant for the cure rate of patients; this appears to be in contrast with results reported by other authors [19], who evidenced high percentages of CD8+ tumor-infiltrating lymphocytes being associated with the absence of lymphnode metastases in cervical cancer. A possible explanation of this discrepancy may depend on the differences of the cases studied; in fact, we investigated the immunoprofile in intraepithelial (preinvasive) lesions, while these authors studied frankly malignant cancers. Results from the present study are consistent with those reported in a previous paper from our group [47], in which we demonstrated a significantly higher distribution of CD4+ T cells in patients without disease recurrence, compared to relapsing disease cases. The relative importance of CD4+ T cells in cervical tissues affected by hr-HPV lesions has also been underlined by other authors [25], who highlighted that lesions spontaneous regression was associated with higher rates of CD4+ T cells infiltrates; our results strengthen these previous observations, adding the important demonstration that CD4+ T cells higher rates positively and significantly influence the outcome of patients after conservative surgical treatment. With regards to the evaluation of T-bet and GATA-3 transcription factors, our results exactly reflected their specific immunological functions: the first is in fact involved in the differentiation of a Th1 immune response which dominates in case of HPV infection, while GATA-3 is involved in the shift towards a Th2 response which is responsible for B cells' activation that surely have less importance against a viral pathogen. Consistently with the CD4+ T cells results, higher T-bet+ immunohistochemical positivity has been demonstrated to significantly correlate with favourable patients' outcome; of note, no cases of disease recurrence were present in the group of patients with both CD4+ and T-bet+ higher distributions in cervical samples. Thus, it is interesting to observe that, in consideration of the predominant role of T-bet transcription factor in viral infections, a significantly higher T-bet+ activity correlated with a better prognosis; this observation is also reinforced by the strong statistical significance obtained from the association tests. On the other hand, no significance has been obtained in terms of prognostic significance of GATA-3 transcription factor; in fact, no differences emerged among patients in terms of clinical outcome. This result may be interpreted as expected, due to the GATA-3+ involvement in the Th2 immune response. In this regard, previous authors [48] performing an immunohistochemical analysis of cervical tissue of patients with different degrees of HPV-related lesions showed that GATA-3 transcription factor was detectable in healthy controls and in low- and intermediate-grade intraepithelial lesions, but was completely absent in high-grade lesions and cervical cancer. The authors therefore, suggested that a down regulation of GATA-3 may be advocated as a factor correlating with the development of cervical carcinogenesis; according to our results, such hypothesis cannot be further commented or discussed, though we believe that, to support this theory, some kind of decrease in GATA-3+ positivity should at least have been detected in relapsing cases. Finally, with regard to innate immunity, our results show that an association between CD11c+ dendritic cells and patients' outcome can be traced (*P* < 0.001 for the stroma). As it can be seen from the statistical analysis, while the positivity within the epithelium does not show a significant association with the outcome of disease, the stromal histochemical positivity showed a statistically significant inverse correlation: in fact the Phi value of −0,63 indicates that high positivity for stromal dendritic cells is associated with a higher frequency of regression of disease. It is known that, due to its immune escape mechanisms, HPV prevents the activation and maturation of dendritic cells, thus preventing the activation of a correct immune response by cytotoxic T-lymphocytes. In addition, in patients with cervical cancer, low distribution or complete absence of dendritic cells in the affected areas is reported [43]. Our results may indicate that the subepithelial increase of dendritic cells observed in regressing cases could be seen as a sort of “escape from the escape” mechanism, limiting the HPV prevention of dendritic cells' activation, and therefore acting as a protective prognostic factor reducing the disease recurrence rates; the reason why this only occurs in a subset of patients is not clear and needs to be confirmed and more extensively studied.

## 5. Conclusions

Results from the present study strongly confirm the evidence that host's mucosal immune response at the level of cervical tissues plays a key role in terms of influencing the natural history of high-grade cervical preneoplastic HPV-related lesions; in particular, we demonstrated a strong correlation between specific immunological tissue phenotypes and clinical outcome after surgical excision of a preneoplastic lesion. CD4+ T cells and T-bet+ transcription factor in the epithelium and CD11c+ dendritic cells in the stroma represent the immunological patterns that, when detected at higher levels of immunohistochemical positivity at the moment of cervical conization, can predict a statistically significative lower frequency of disease recurrence.

## Figures and Tables

**Table 1:** Positivity of immunohistochemical targets within the epithelium.

	NED		REC			
	<5%	>5%	<5%	>5%	Fisher exact	Phi
CD4+	6 (27.3%)	16 (72.7%)	12 (100%)	0 (0%)	**<0.0001**	−0.70
CD8+	19 (86.4%)	3 (13.6%)	10 (83.3%)	2 (16.7%)	n.s.	0.04
T-bet+	4 (18.2%)	18 (81.8%)	12 (100%)	0 (0%)	**<0.0001**	−0.78
GATA3+	22 (100%)	0 (0%)	10 (83.3%)	2 (16.7%)	n.s.	0.34
CD11c+	11 (50%)	11 (50%)	10 (83.3%)	2 (16.7%)	n.s.	0.22

NED: not evidence of disease; REC: recurrence of disease.

**Table 2:** Positivity of immunohistochemical targets within the stroma.

	NED		REC			
	<5%	>5%	<5%	>5%	Fisher exact	Phi
CD4+	3 (13.6%)	19 (86.4%)	12 (100%)	0 (0%)	**<0.0001**	−0.83
CD8+	13 (59.1%)	9 (40.9%)	10 (83.3%)	2 (16.7%)	n.s.	−0.25
T-bet+	3 (13.6%)	19 (86.4%)	12 (100%)	0 (0%)	**<0.0001**	−0.83
GATA3+	14 (63.6%)	8 (36.4%)	7 (83.3%)	5 (41.7%)	n.s.	0.05
CD11c+	4 (18.2%)	18 (81.8%)	10 (83.3%)	2 (16.7%)	**0.0001**	−0.63

NED: not evidence of disease; REC: recurrence of disease.
